# Modified donor blood flow-preserved cross-leg anterolateral thigh flap procedure for complex lower extremity reconstruction

**DOI:** 10.1186/s13018-022-03155-9

**Published:** 2022-05-12

**Authors:** Hong-Xiang Zhou, Liang He, Dong Yin, Yang Niu, Zhe Jin, Jun-Jie Li, Qian-Kun Wang, Tao Zhou

**Affiliations:** 1 Department of Orthopedics, The First Affiliated Hospital of Anhui Medical University, No. 218, Jixi Road, Hefei, 230022 Anhui China; 2 Department of Orthopedics, The Fuyang People’s Hospital of Anhui Medical University, Anhui, China

**Keywords:** Cross-leg anterolateral thigh flap, Flow-through, Free skin wrapping of the vascular pedicle, Microanastomosis

## Abstract

**Background:**

Complex lower limb reconstruction due to severe trauma remains a challenge for reconstructive surgeons. Here, we introduce a modified donor blood flow-preserved cross-leg anterolateral thigh flap procedure and evaluate its clinical efficacy.

**Methods:**

Between January 2013 and December 2019, 22 patients (range 10 to 64 years old) with unilateral lower limb injury underwent modified donor blood flow-preserved cross-leg anterolateral thigh flap procedures. Among them, 16 cases were traffic accidents, 5 cases were persistent ulcers, and 1 case was a degloving injury. The arterial pedicle of the flap was prepared in a Y-shaped fashion and microanastomosed to the posterior tibial artery of intact leg in a flow-through style. A split-thickness skin graft was applied to wrap the vascular pedicle after anastomosis. The flap was designed in a single or bilobed fashion according to the shape of the tissue defect. The operation time, the intraoperative blood loss and the length of hospital stays were recorded. The vascular pedicle was divided 4 weeks after anastomosis. Doppler ultrasound was performed to evaluate the blood flow of the donor posterior tibial artery during postoperative follow-up.

**Results:**

All 22 flaps survived. The tissue defects ranged from 12 × 6 to 21 × 18 cm^2^. The flap sizes ranged from 14 × 7.5 to 24 × 21 cm^2^. The average operation time, intraoperative blood loss and length of hospital stays were 6.73 ± 1.49 h, 280.95 ± 59.25 ml and 30.55 ± 2.52 days, respectively. Eighteen flaps were designed in a single fashion, while four were in bilobed fashion. Twenty patients underwent fasciocutaneous flap transplantations, while two underwent musculocutaneous flap transplantations. Two cases developed local lysis of the flap which healed after further debridement. Direct suture of the incision after flap harvest was performed in 16 cases, while additional full-thickness skin grafting was performed in the remaining 6 cases. Further bone transport procedures were performed in 15 patients who had severe tibia bone defects. The blood flow of donor posterior tibial artery was confirmed in all patients during follow-ups. All patients recovered flap sensation at the final follow-up. The postoperative follow-ups ranged from 18 to 84 months, and no long-term complications were observed.

**Conclusions:**

The modified donor blood flow-preserved cross-leg anterolateral thigh flap procedure is an ideal method to repair severe lower limb trauma with tibial artery occlusion which avoids sacrificing the major artery of the uninjured lower limb.

## Background

Complex lower extremity injury due to high-energy trauma remains a severe clinical challenge to date. The severe lower limb trauma is frequently characterized by compromised main vessels and extensive soft tissue defects, which are always accompanied by other conditions, such as bone exposure, bone defect, and infection. In such situations, local pedicled flap strategies are unachievable due to poor surrounding soft tissue. In addition, free flap transfers within the injured lower limb are also impossible because the donor vessels in the ipsilateral leg are unavailable. Cross-leg pedicled flaps are reliable choices to salvage traumatized lower limb with both complex soft tissue and vessels damages. Since Hamilton first introduced it in 1854, cross-leg pedicled flap method has been widely used in complex lower extremity reconstruction, and various cross-leg pedicled flap procedures have been reported for various degrees of lower limb injury [1, 2]. The common feature of these procedures is that the pedicled flap is designed based on the major arteries in intact lower limb, mainly including the posterior tibial artery and the peroneal artery [3–9]. Although cross-leg pedicled flaps have a favorable survival rate of nearly 100%, its disadvantages are also apparent; for example, limited coverage of the tissue defects, the sacrifice of tissues or vessels in healthy side, and aesthetic impairment of the healthy leg [2].

Since the advent of microsurgery technology, microsurgical free flap transfer has gradually become the preferred strategy for complex lower extremity reconstruction. When there is no available donor artery in the injured lower limb, cross-leg free flap procedures are optional methods [10]. In this procedure, the free flap is temporarily nourished by suitable donor arteries of the contralateral lower limb. When the flap is adequately revascularized, the vascular pedicle is separated from the flap. To ensure a high survival rate of the free flap, major arteries in the contralateral leg are always chosen as the donor arteries, and end-to-end micro-anastomosis is preferred. The main disadvantage of this procedure is that the blood supply to the distal lower limb is obviously reduced in healthy side. A cross-leg flow-through pedicled free flap procedure is established, which preserves the integrality of donor artery and blood circulation in healthy lower limb [11–13]. In this promising procedure, the arterial pedicle is prepared in a reverse Y-shaped fashion and further inserted into the donor artery by anastomosing the Y-shaped arterial branches to the two transected ends of the donor artery. However, Y-shaped arterial bifurcation is not always available. In addition, postoperative management of the pedicle is also difficult.

Surgical treatments are difficult for severe lower limb trauma with both extensive soft tissue defects and only one surviving main artery. Hence, in this study, we introduced a modified donor blood flow-preserved cross-leg anterolateral thigh flap procedure to reconstruct the severely damaged lower limb. By presenting our clinical outcomes of the modified procedure, we aim to further popularize the clinical application of the cross-leg free flap surgery.

## Methods

### Patients

A retrospective study was conducted on patients who suffered from severe unilateral lower limb trauma and underwent the modified donor blood flow-preserved cross-leg anterolateral thigh flap procedure in our hospital between January 2013 and December 2019. The inclusion criteria were patients with both extensive soft tissue defects distal to the knee joint in one lower limb and at least one tibial artery occlusion (anterior tibial artery (aTA) or posterior tibial artery (pTA)). The exclusion criteria were patients with (1) vascular injuries in the contralateral lower limb, (2) poor control of hypertension (blood pressure over 140/90 mmHg), (3) poor control of diabetes (fasting blood-glucose over 7 mmol/L or glycosylated hemoglobin over 7%), (4) smoking, and (5) deep vein thrombosis of lower limb. A total of 22 consecutive patients were included. There were 17 males and 5 females. The average age was 37 years old (range, 10–64 years old). Among them, 16 cases were admitted or transferred to our emergency department due to traffic accidents, 5 cases were admitted to our outpatient department due to stubborn ulcers, and 1 case was admitted to our emergency department due to a degloving injury. Among the 16 traffic accident patients, one had an injury at the dorsum pedis, the rest had severe open tibia fractures, including 7 Gustilo IIIB injuries and 8 Gustilo IIIC injuries [14]. All 5 persistent ulcers were caused by previous trauma (range of disease duration, 3–10 years). Twelve patients sustained left-side lower limb injuries, and ten patients sustained right-side injuries. Among all the cases, 19 involved the crus, 2 involved the medial malleolus, and 2 involved the dorsum pedis. The wound was thoroughly debrided for emergency patients and covered with vacuum sealing drainage devices (VSD devices). Temporary internal or external fixations of the fractures were performed, if necessary. When patients’ vital signs were stable, and the infection was controlled, free flap transplantation was performed. For nonemergency patients, a flap procedure was performed after effective control of the wound infection. Before flap transplantation, a computed tomography angiography (CTA) was routinely performed to evaluate the circulation of the injured lower limb. In this study, all patients had major arteries damaged in the injured lower limb, and 86% of them had two or more arteries compromised. These arteries included the peroneal artery (PA), aTA, pTA, and dorsalis pedis artery (DPA). Among all the patients, 4 had hypertension, and 1 had diabetes. Detailed demographic information on each patient was shown in Table 1.

**Table 1:** The demographic and surgical details of the patients

Case	Sex	Age	Injury type	Injury location	Soft tissue defect (cm^2^), fracture status	Artery injury	Comorbidities	Flap type	Flap area (cm^2^)	Complica-tion	Second procedure
1	F	55	Traffic accident	Crus (R)	18 × 15, Gustilo IIIC	PA, aTA and pTA	Hypertension	Single	22 × 17	/	Ilizarov
2	M	55	Stubborn ulcer	Crus (R)	24 × 14	aTA and pTA	/	Single	27 × 16	/	/
3	M	33	Traffic accident	Knee and Crus (L)	27 × 13, Gustilo IIIC	PA, aTA and pTA	/	Single	30 × 16	/	Ilizarov
4	M	29	Traffic accident	Crus (R)	22 × 11, Gustilo IIIC	PA, aTA and pTA	/	Bilobed	25 × 12	/	Ilizarov
5	M	24	Degloving injury	Crus and foot (L)	22 × 16 (entire planta)	aTA and pTA	/	Single	24 × 19	/	/
6	M	10	Traffic accident	Crus (R)	22 × 15, Gustilo IIIC	PA, aTA and pTA	/	Single	24 × 18	/	Ilizarov
7	M	26	Stubborn ulcer	Medial malleolus (L)	14 × 8	pTA	/	Bilobed	17 × 8	/	/
8	M	20	Traffic accident	Crus (L)	17 × 10, Gustilo IIIB	PA and aTA	/	Single	19 × 12	/	Ilizarov
9	F	18	Traffic accident	Crus (R)	20 × 14, Gustilo IIIC	PA, aTA and pTA	/	Single	23 × 17.5	Local lysis	debridementand Ilizarov
10	M	60	Traffic accident	Crus (R)	20 × 12, Gustilo IIIB	aTA and pTA	/	Single	24 × 15	/	Ilizarov
11	M	48	Traffic accident	Dorsum pedis (L)	21 × 18	aTA and DPA	Hypertension	Single	24 × 21	/	/
12	M	64	Traffic accident	crus (L)	12 × 8, Gustilo IIIB	aTA and pTA	Diabetes	Single	14 × 10	/	Ilizarov
13	M	23	Traffic accident	Crus (L)	15 × 10, Gustilo IIIB	aTA and pTA	/	Bilobed	22 × 9	/	Ilizarov
14	M	38	Traffic accident	Crus (L)	26 × 13, Gustilo IIIC	PA, aTA and pTA	/	Single	30 × 15	/	Ilizarov
15	F	42	Traffic accident	Crus (L)	17 × 11, Gustilo IIIB	aTA and pTA	/	Single	19 × 14	Local lysis	debridement and Ilizarov
16	M	37	Traffic accident	Crus (R)	18 × 10, Gustilo IIIC	PA, aTA and pTA	/	Single	21 × 12	/	Ilizarov
17	M	21	Traffic accident	Crus and dorsum pedis (L)	15 × 9, Gustilo IIIB	aTA, pTA and DPA	/	Single	17 × 11	/	Ilizarov
18	M	44	Stubborn ulcer	crus (L)	15 × 8	aTA	/	Single	17 × 10	/	/
19	F	46	Stubborn ulcer	Medial malleolus (R)	12 × 6	pTA	/	Single	14 × 7.5	/	/
20	M	60	Stubborn ulcer	Crus (L)	17 × 12	PA and pTA	hypertension	Single	20 × 14	/	/
21	M	22	Traffic accident	Crus (R)	22 × 12, Gustilo IIIC	PA, aTA and pTA	/	Bilobed	26 × 13	/	Ilizarov
22	F	46	Traffic accident	Crus (R)	12 × 10, Gustilo IIIB	aTA and pTA	hypertension	Single	15 × 13	/	Ilizarov
	M (77%)	10–64	Traffic accident (73%)	Crus (86%), L (55%)	12–27 × 6–18	Two or more (86%)		Single (82%)	14–30 × 7.5–21	Local lysis (9%)	Ilizarov (68%)

*M* male, *F* female, *PA* peroneal artery, *aTA* anterior tibial artery, *pTA* posterior tibial artery, *DPA* dorsalis pedis artery, *Ilizarov* Ilizarov technique, patients were listed by admission time

This study was approved by the Ethics Committee of the First Affiliated Hospital of Anhui Medical University. All patients signed the informed consent form for clinical data publication. All procedures were in compliance with the tenets of the Helsinki Declaration.

### Surgical procedure

**Fig. 1:**
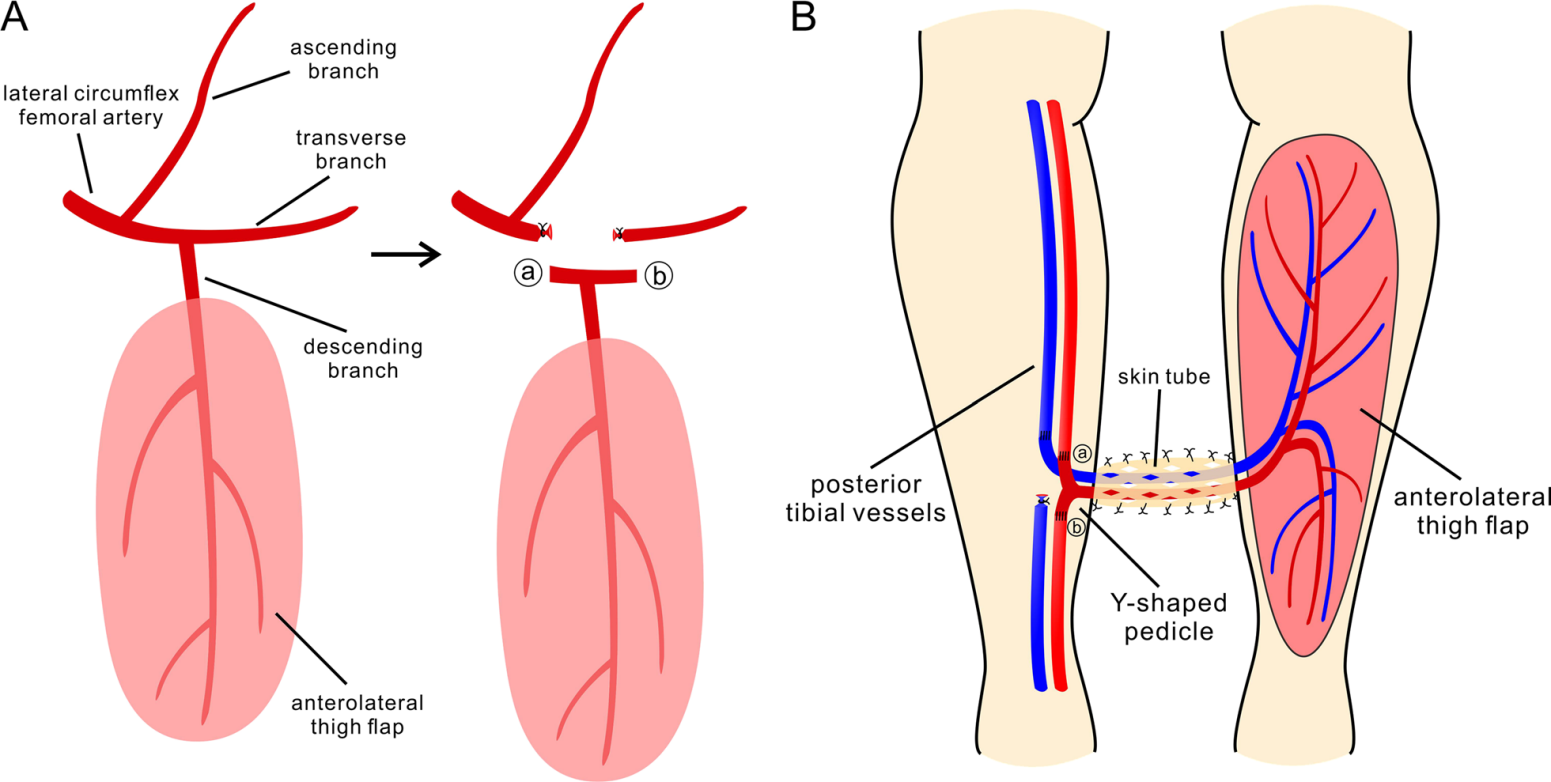
**a**, **b** Illustration of the modified recipient blood flow-preserved cross-leg anterolateral thigh flap procedure

In this study, all procedures were performed by a same surgeon (the first author, Dr. Zhou). All patients underwent the modified donor blood flow-preserved cross-leg free flap transplantation using anterolateral thigh fasciocutaneous or musculocutaneous flaps (Fig. 1). Briefly, under general anesthesia, VSD devices or fracture fixation devices were removed. Thorough debridement of necrotic tissue was performed. The edge of the tissue defect was trimmed, and the defect size was measured. According to the size and shape of the tissue defect, we designed a suitable anterolateral thigh flap, which was approximately 2–3 cm longer in length and width than the defect. The flap was chosen on the same side of the injured leg if the patient had no special requirement. According to the methods introduced in previous literature, a standard anterolateral thigh flap based on the lateral circumflex femoral arterial system was harvested [15, 16]. The descending branch of the lateral circumflex femoral artery was dissected proximally between the rectus femoris muscle and the vastus lateralis muscle. At the proximal arterial bifurcation, the lateral circumflex femoral artery and its transverse branch were also exposed to a length of approximately 2 cm. This arterial bifurcation was prepared in a Y-shaped fashion. The descending arterial branch and its accompanying veins were prepared as the vascular pedicle and divided from the lateral circumflex femoral vascular system with a sufficient length of approximately 8–10 cm. In addition, the flap was prepared in a single or bilobed fashion according to the defect shape. Then, the posterior tibial vessels and the great saphenous vein of the uninjured lower limb were dissected and prepared as the donor vessels. After the insertion of the flap into the tissue defect, the two arterial ends of the Y-shaped pedicle (lateral circumflex femoral artery inlet and transverse branch outlet) were micro-anastomosed to the proximal and distal transected ends of the posterior tibial artery, respectively. Two comitant veins were also reconstructed microsurgically. After confirming the blood reperfusion of both the flap and the distal posterior tibial artery, a split-thickness skin graft was applied to wrap the vascular pedicle protectively. Finally, the two legs were fixed in a parallel position by cross external fixation devices. The fixed distance between two legs was adjusted according to the length of the vascular pedicle, which should make the pedicle completely free of tension. The operation time and the intraoperative blood loss were recorded. Three weeks later, we assessed the revascularization of the flap by intermittent pedicle clamping for approximately one week. After confirmation of flap revascularization, the vascular pedicle was transected and ligated, and the flap was separated from the donor leg. The cross external fixation devices were also removed, and a physiotherapy program was conducted after surgery. The length of hospital stays was recorded. In addition, for patients with large segmental tibia bone defect, a bone lengthening procedure (Ilizarov technique) was performed 4 weeks after pedicle division according to the methods reported in previous literature [17–19].

Three to six months after pedicle division, a color Doppler ultrasound was performed to evaluate the blood flow of the posterior tibial artery in donor side.

## Results

In this study, the contralateral posterior tibial artery was chosen as the donor artery in all patients. The defect sizes of the injured lower limb ranged from 12 to 27 cm in length and 6 cm to 18 cm in width (mean length × width, 18.5 cm^2^ × 11.6 cm^2^). The sizes of the anterolateral thigh flap ranged from 14 to 30 cm in length and 7.5 cm to 21 cm in width (mean length × width, 21.5 cm^2^ × 13.6 cm^2^). The flap was designed in a single fashion in 18 cases and a bilobed fashion in 4 cases. Twenty patients underwent fasciocutaneous flap transplantation, and the remaining two (case 9 and case 14), who had deep dead space in the defect site, underwent musculocutaneous flap transplantation. The average operation time was 6.73 ± 1.49 h. The average intraoperative blood loss was 280.95 ± 59.25 ml. In postoperative dressing change, the vascular pedicle was protected by embedding petrolatum gauze pieces. All 22 flaps survived uneventfully. Among all the patients, two experienced postoperative local lysis of the flap (case 9 and case 15), and the wound healed after further debridement and positive dressings. There were 16 patients who underwent direct suture of the incision after flap harvest, and the remaining 6 underwent full-thickness skin grafting after partial suture. In all patients, no complications occurred after suture. The average length of hospital stays was 30.55 ± 2.52 days. Fifteen patients with severe tibia bone defects underwent further bone lengthening procedure after flap transplantation. Anterograde blood flow of the donor posterior tibial artery was confirmed using the Doppler ultrasound test in all patients during follow-ups. Six months after flap surgery, the flap senses of touch and pain gradually recovered. At the last follow-up, all flaps recovered sensation. The appearance, including color and texture, was close to the surrounding normal sites in all flaps. All patients returned to their daily work without flap-related discomfort. Overall, all patients were satisfied with the clinical results, and no complications were observed during the long-term follow-up (range, 18 to 84 months).

**Fig. 2:**
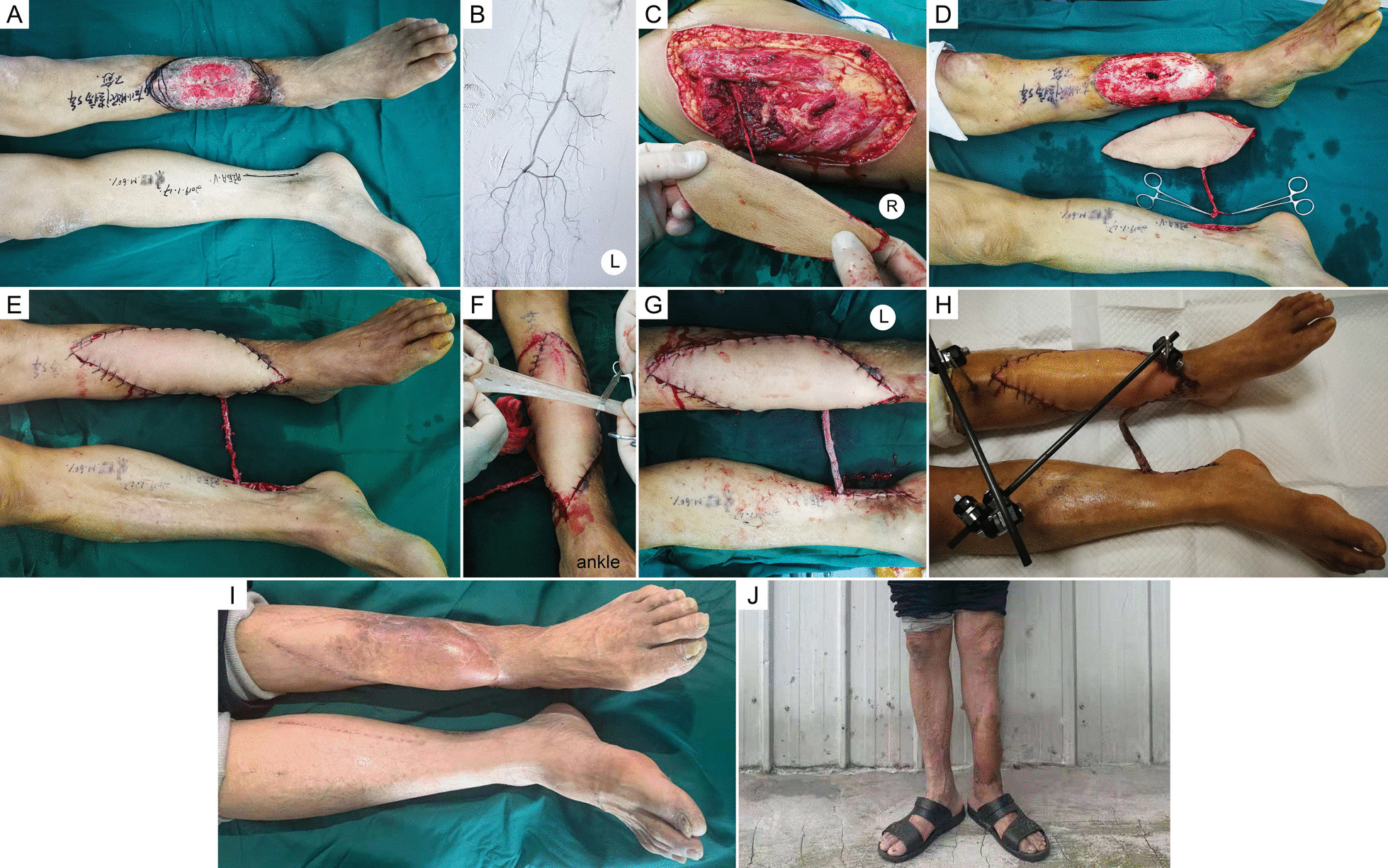
Case 20, a 60-year-old man. **a** A persistent ulcer in the left crus for 5 years. **b** Preoperative CTA showed that only the anterior tibial artery was unobstructed in the left crus. **c** An anterolateral thigh flap was harvested. **d** The defect size was 17 × 12 cm^2^ after thorough debridement. **e** Modified cross-leg flow-through flap transplantation was performed. **f, g** A meshed split-thickness skin was prepared and used to wrap the vascular pedicle. **h** Four days after flap procedure. **i, j** Twenty months after the operation

**Fig. 3:**
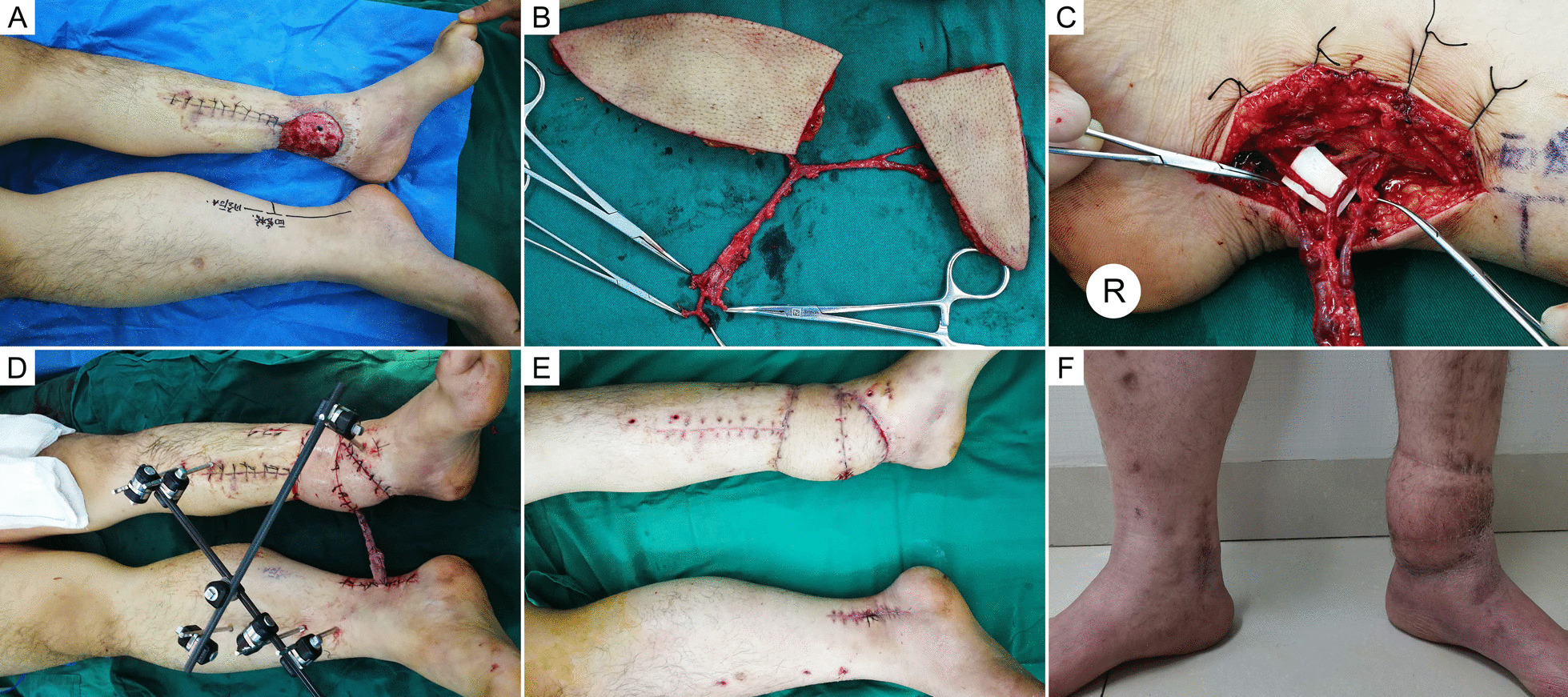
Case 7, a 26-year-old man. **a** A persistent ulcer in the left medial malleolus for 4 years. **b** A bilobed flap was prepared. **c** The arterial pedicle was prepared in a Y-shaped fashion and microanastomosed to the contralateral posterior tibial artery in a flow-through style. Two comitant veins were also microanastomosed. **d** The modified cross-leg free flap procedure was performed. **e** The vascular pedicle was separated 4 weeks after flap transplantation. **f** Seven years after the operation

**Fig. 4:**
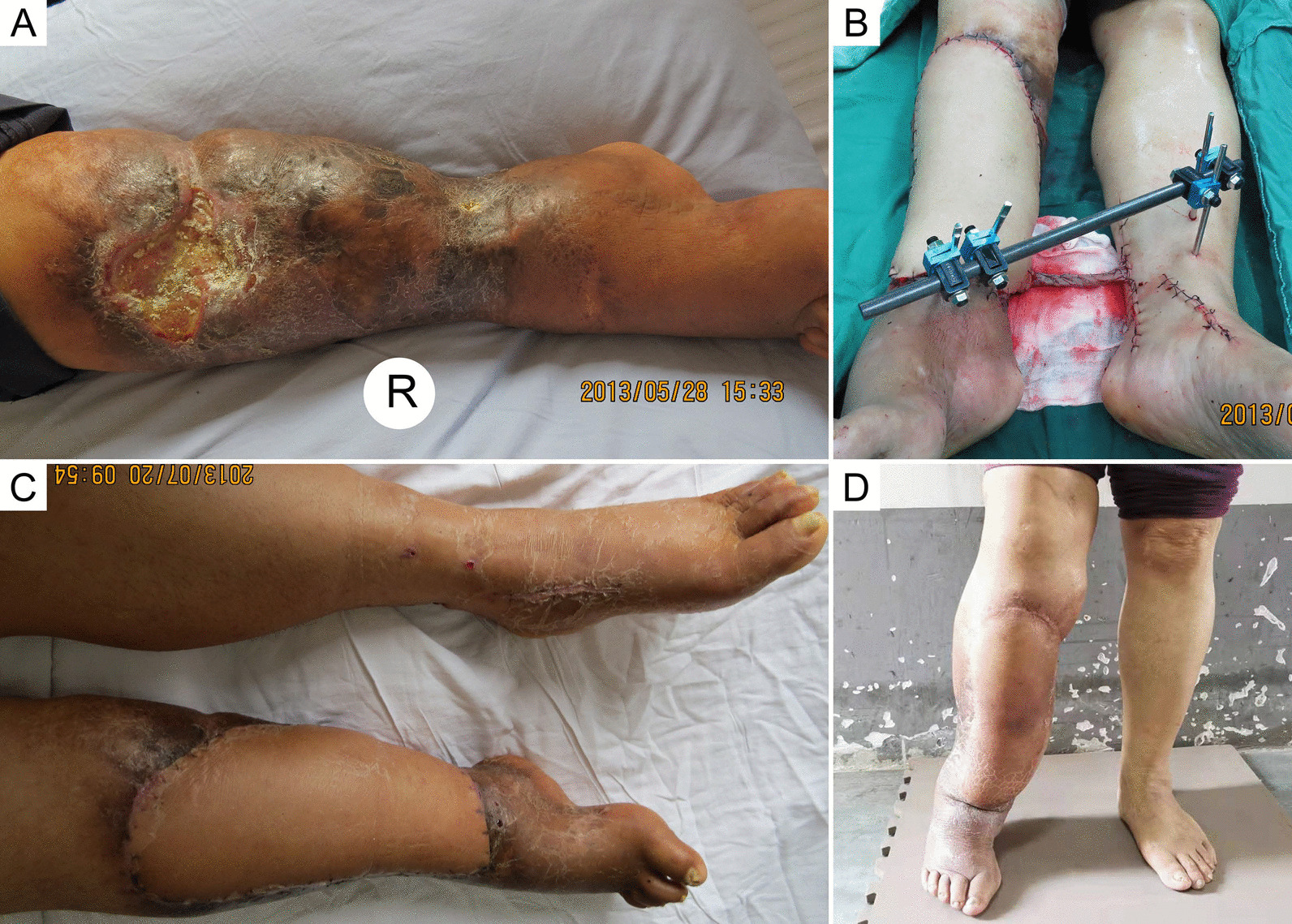
Case 2, a 55-year-old man. **a** A persistent ulcer in the right crus for 8 years. **b** The modified cross-leg free flap procedure was performed. **c, d** Seven years and ten months after the operation

**Fig. 5:**
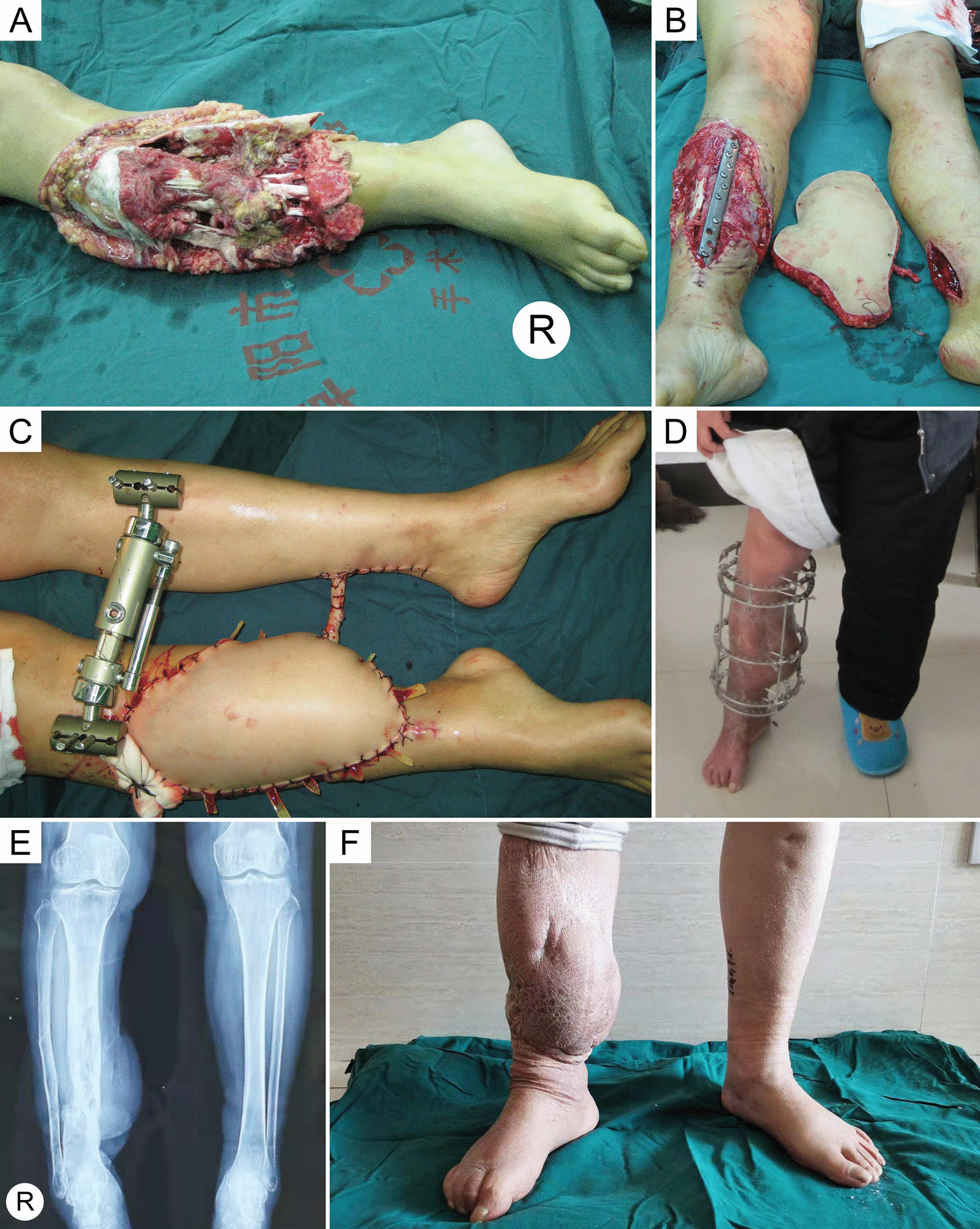
Case 1, a 55-year-old woman. **a** Gustilo IIIC trauma in the right crus. **b** The defect size was 18 × 15 cm^2^ after thorough debridement. **c** The modified cross-leg free flap procedure was performed. **d** Fifteen months after pedicle division. **e**, **f** Eight years after the operation

Typical cases are presented in Figs. 2, 3, 4, and 5. Detailed information is shown in Table 1.

## Discussion

Severe lower limb trauma with both large-sized soft tissue defects and tibia bone exposure is frequently encountered at the emergency department, and further reconstruction remains a major challenge for surgeons [20]. In this situation, flap procedures were necessary for defect coverage and limb salvage. However, the local flap technique or ipsilateral free flap transplantation is often impossible due to extensive tissue defects and potential vascular damage. In this study, we introduced a modified donor blood flow-preserved cross-leg anterolateral thigh flap procedure to reconstruct complex lower limb injuries. This method is an effective and safe alternative for patients with different sizes of tissue defects in the unilateral lower limb.

The cross-leg flap technique has long been used in patients with lower limb injuries. Since Stark first introduced it in 1952, various cross-leg flap procedures have been reported, including pedicled flap procedures and free flap procedures [1, 21]. For small-sized tissue defect, the cross-leg pedicled flap procedure is preferred because it is relatively easy and safe to perform [22]. However, for large tissue defect, the cross-leg free flap procedure is the optimal strategy [23, 24]. Cross-leg free flap surgery has been applied to lower limb reconstruction since 1979 [25]. In this procedure, the free flap was temporarily nourished by vessels of the uninjured lower limb and was separated from the intact leg after flap revascularization. Skilled microsurgical techniques are requisite for this procedure. With the increasing popularity of microsurgical technique, free flap transplantation has gradually become an important method for salvaging severely traumatized lower limb [23, 26, 27]. A favorable lower extremity salvage rate of 93% was reported in free flap transplantation [27]. Compared with an end-to-side fashion, an end-to-end anastomosis between vascular pedicle and donor vessels is preferred in the free flap procedure, which obviates potential thrombosis caused by change in blood flow at the anastomosis site. However, the major drawback of end-to-end anastomosis is sacrificing the donor artery, which results in a considerable reduction in blood supply to the distal leg. In early-stage clinical studies of the cross-leg free flap technique, donor arterial sacrifice was considered inevitable [28]. Nevertheless, in recent years, several innovative techniques have been reported to preserve the integrity of the donor artery. In 2000, Topalan et al. introduced a latissimus dorsi free flap with a Y-shaped arterial pedicle in lower limb construction. This strategy not only provided blood supply to the flap but also preserved the blood flow of the donor artery; it is called the flow-through technique [13, 29]. For the case without arterial bifurcation, Akyurek et al. introduced a two-stage method to restore the continuity of the donor artery in 2002 [30]. In the first stage, an end-to-end anastomosis was performed between the thoracodorsal artery of the latissimus dorsi free flap and the uninjured donor posterior tibial artery. After flap revascularization, the thoracodorsal artery was transected and rerouted to the distal end of the posterior tibial artery in the second stage. In 2016, Gencel et al. performed the cross-leg flow-through pedicled latissimus dorsi free flap procedure on 6 patients with high voltage electrical injuries [11]. In this small-sample clinical study, the thoracodorsal artery and circumflex scapular artery (or serratus branch) were prepared as T-shaped pedicles and further anastomosed to the contralateral posterior tibial artery. Adequate diameter and blood flow of the posterior tibial vascular system contribute to the survival of the flap. In 2020, Bali et al. conducted a study of 12 patients who underwent donor blood flow-preserved cross-leg free flap procedure [12]. In Bali’s study, an anterolateral thigh fasciocutaneous flap was used in 8 patients to reconstruct small or medium defects, while a latissimus dorsi flap was used in 4 patients with large defects. In addition, the arterial pedicle was designed in a T-shaped fashion in the anterolateral thigh flap but not in the latissimus dorsi flap. Thus, the blood flow of the donor posterior tibial artery was reestablished in the first stage of the anterolateral thigh flap procedure (flow-through technique) and in the second stage of the latissimus dorsi flap procedure (rerouting technique). To our knowledge, there are only a few studies of flow-through pedicled cross-leg free flap technique for lower limb reconstruction, all of which involved a small number of cases [11, 12, 29, 31–34]. In these studies, flaps were chosen based on the size and shape of the tissue defect. Generally, the anterolateral thigh flap is preferred for relatively small defect, while the latissimus dorsi flap is suitable for relatively large defect. Compared with the anterolateral thigh flap, the latissimus dorsi flap is relatively traumatic to harvest, and it is difficult to prepare a Y-shaped arterial pedicle. In addition, the surgical position needs to be changed during cross-leg latissimus dorsi flap transplantation. Therefore, the anterolateral thigh flap is ideal for complex lower limb reconstruction, but it is hindered in popularity due to limited defect coverage. Traditionally, the proximal part of the free flap is used to cover the vascular pedicle, which will reduce the effective flap area for defect coverage. In the present study, we made two modifications as follows: (1) the lateral circumflex femoral arterial bifurcation was employed to design the Y-shaped arterial pedicle instead of the perforating arterial branches; (2) the vascular pedicle was wrapped by split-thickness skin instead of the proximal part of the flap. We performed this modified flow-through pedicled cross-leg free flap procedure on patients with different sizes of tissue defect. The main indication is severe trauma or a large stubborn ulcer in unilateral lower limb, which requires free flap transplantation, but no suitable donor artery was available in the injured leg. The contraindications include pTA injury in the contralateral lower limb, coagulation disorders, or conditions in which patients are intolerant to surgery.

Compared to previous techniques, our method has several advantages. First, meshed split-thickness skin was used to wrap the vascular pedicle. For anterolateral thigh flap, a sufficient length of vascular pedicle can be harvested. Instead of flap coverage, we performed a free skin wrapping technique to protect the vascular pedicle. On the one hand, the obtained flap can be fully used for defect coverage, which means that the anterolateral thigh flap procedure is suitable for patients with large-sized tissue defect. Our study suggests that various defects ranging from 72 to 378 cm^2^ could be effectively covered by relevant-sized anterolateral thigh flap. On the other hand, compared with flap coverage, skin wrapping is more convenient for postoperative blood flow observation and dressing change, and it is much easier to perform pedicle division in the second-stage procedure [12]. The free skin wrapping technique has been reported previously [35]. Serel et al. reported a case of an unhealed wound on the right foot that underwent a cross-leg anterolateral thigh perforator flap procedure in which split-thickness skin was used to wrap the vascular pedicle [35]. Together with our results, meshed split-thickness skin grafting is an effective and convenient method for vascular pedicle coverage.

Second, the lateral circumflex femoral arterial system is employed for the design of the Y-shaped arterial pedicle. The lateral circumflex femoral artery consistently gives off the transverse branch and descending branch. This arterial bifurcation is not difficult to dissect and prepare to a Y-shaped fashion. The anterolateral thigh free flap is widely used in posttraumatic reconstruction, including reconstruction of the upper limb, lower limb, and body trunk [15, 36, 37]. The anterolateral thigh flap can be harvested as a fasciocutaneous flap or a musculocutaneous flap for the reconstruction of different injury types. Generally, a satisfactory survival rate and aesthetic outcome can be achieved by the anterolateral thigh flap procedure [36]. In addition, the anterolateral thigh flap is rich in its arterial perforator system, which is beneficial to flap survival on the one hand and can be designed as a single style or a bilobed style on the other hand. A bilobed flap is an optimal and practical choice to cover irregular tissue defect. In particular, when the defect width is large, a large-length single anterolateral thigh flap can be redesigned as a large-width bilobed flap. In our case series, both single and bilobed flap procedures produced good clinical results.

Third, harvesting anterolateral thigh flap and transplanting cross-leg flap are performed in the same surgical position, which can shorten the first-stage operation time. Furthermore, the vascular pedicle is easily separated after flap revascularization, which shortens the second-stage operation time. After flap transplantation, parallel external fixation between the two legs is relatively comfortable.

One disadvantage is that the incision after flap harvest is relatively large; hence, direct suture could not be practicable in some patients. In this situation, a second-stage skin grafting is necessary. Another disadvantage is that two anastomoses increase the risk of postoperative vascular occlusion. Skilled microsurgery techniques are necessary.

There are some limitations of our study. Postoperative parallel fixation of both lower limbs is necessary, and the fixation time is relatively long (4 weeks). Pressure ulcer prevention and physical therapy intervention should be carried out during the fixation period. In our procedure, the incision after flap harvest was relatively large, some cases required further skin grafting (27% in our study). This study was a retrospective design and a relatively small sample size. Hence, a multicenter large-sample prospective study is needed in the future.

## Conclusion

In conclusion, the modified donor blood flow-preserved cross-leg anterolateral thigh flap procedure is an effective and promising technique for complex lower extremity reconstruction. This technique is an ideal method for repairing large tissue defect without sacrificing the major artery of the uninjured lower limb.

## Data Availability

Not applicable.
